# Cases Reported by Wm. Herbert Lowe, D.V.S.

**Published:** 1883-10

**Authors:** 


### CASES REPORTED BY WILLIAM HERBERT LOWE, D.V. S., FROM DR. MUSTOE'S WILLIAMSBURGH VETERINARY INFIRMARY.

### IV.—THE SUPERIOR PORTION OF THE OS PEDIS REMOVED FROM A DISEASED FOOT, AND THE HORSE SERVICEABLE FOR TWO YEARS AFTER THE OPERATION.

The subject was a large iron gray gelding, *aet* eight years; the property of the Waterbury Cordage Co., of Brooklyn. Two years ago this horse suffered from a punctured wound in the near hind foot, caused by the animal stepping on a nail. The horse was treated by Dr. J. F. Mustoe. The orifice of the wound was enlarged and the usual antiseptic and astringent therapeutic measures employed. Suppuration set in and under ran the frog and a large portion of the sole, which parts were removed. Soon symptoms of necrosed bone developed which lead to the belief that a portion of the bone would have to be removed. A large portion of the bone did become detached and was removed from the plantar surface near the frog. On examination the piece proved to be the entire superior portion of the os pedis.

The possibility of this portion of the bone coming away can be readily understood, but how the animal could recover and be of service for two years after the operation, is much harder to account for. It is a pathological fact that horses are more disposed to develope abnormal bony growths than other animals. The exostosis of spavins, splints, ringbones, etc., in the horse are illustrative of this peculiar diathesis in the equine patient.

In this case a bony deposit took place all around the pastern in the seat of ringbone. Anchylosis must have taken place at the first and second phalangeal articulations, but where did the tendon of the extensor pedis muscle insert itself. The horse was sold to a farmer and although he had a club-foot, yet, as stated above, he was service able for two years when he contracted Glanders and was destroyed. We regret in not hearing of the animal's death until several weeks after it had occurred as we wished to prepare a pathological specimen of this foot for the Museum of the Columbia Veterinary College. The portion of bone which came away during life is now in Dr. Mustoe's cabinet. We would be pleased to have the views that other veterinarians may hold, advanced as to the anatomical and pathological changes that took place in the various tissues of the foot in question.

---

### V.—TYMPANITIS TERMINATING IN RUPTURE OF THE STOMACH.

---

A mare, the property of Cross, Austin & Co., of Brooklyn, was brought to the Williamsburgh Veterinary Infirmary, on the evening of September 10th, suffering from a severe attack of tympanitis. No history was obtainable except that the mare had been sick for about three hours and that no medicine of any kind had been given. The animal was put at once in a spacious box-stall. Vulvitis as well as tympanitis was present. A diagnostic symptom showed itself at once—a regurgitation of food, first from the nostrils and afterwards from the mouth and other symptoms to justify the diagnosis of rupture of the stomach caused by the excessive gases generated not being neutralized.

Unfavorable prognosis given.

*Treatment.*—The regurgitation of food having taken place, paracentesis abdominal is was not performed as it otherwise would have been. Anodynes were exhibited, also remedies to neutralize the gases. Attended patient all night and succeeded in overcoming all the tympanitic symptoms and the vulvitis. Death occurred the following morning at about 10 o'clock.

*Autopsy* was made shortly after death which sustained the diagnosis. On opening the abdominal cavity partially digested oats and other ingesta were found among the viscera; no lesions in the thoracic cavity. Diaphragm in normal condition.

---

### VI.—REMOVAL OF A CYST FROM THE INFERIOR CERVICAL REGION OF A POINTER.

---

The patient was a valuable Pointer; the property of Edmund Orgill, Esq., of Brooklyn. About a year ago a large cyst developed itself in the infèrior cervical region. This cyst was enucleated by Dr. J. F. Mustoe. When opened there escaped a peculiar dark, thick, mucilaginous fluid. The opposing edges of the integument being brought together sutures were put in and the wound healed up rapidly. About six months afterwards an enlargement appeared in the same region, and at the request of Mr. Orgill, the dog was operated upon again. This time the fluid was allowed to escape and a seton was passed through the cyst. The usual antiseptic treatment was adopted and the animal was well again in due time. The cyst developed the third time. The dog being a favorite with the owner, another operation was performed.

A longitudinal incision was made and the fluid allowed to escape which was of the same character as the first. A considerable portion of the skin of either side was removed and the edges of the opposing integument brought together and secured by sutures. The wound healed up rapidly. Dog was sent to the country. No sign of its re-development.

